# Protective effects of the postbiotic deriving from cow’s milk fermentation with *L. paracasei* CBA L74 against *Rotavirus* infection in human enterocytes

**DOI:** 10.1038/s41598-022-10083-5

**Published:** 2022-04-15

**Authors:** Cristina Bruno, Lorella Paparo, Laura Pisapia, Alessia Romano, Maddalena Cortese, Erika Punzo, Roberto Berni Canani

**Affiliations:** 1 Department of Translational Medical Science, University of Naples Federico II, Naples, Italy; 2 ImmunoNutritionLab at CEINGE Advanced Biotechnologies, University of Naples Federico II, Naples, Italy; 3 Institute of Genetics and Biophysics, CNR, Naples, Italy; 4 European Laboratory for the Investigation of Food-Induced Diseases, University of Naples Federico II, Naples, Italy; 5 Task Force for Microbiome Studies, University of Naples Federico II, Naples, Italy; 6 Task Force for Nutraceuticals and Functional Foods, University of Naples Federico II, Naples, Italy

**Keywords:** Cell biology, Gastroenterology

## Abstract

*Rotavirus* (RV) is the leading cause of acute gastroenteritis-associated mortality in early childhood. Emerging clinical evidence suggest the efficacy of the postbiotic approach based on cow’s milk fermentation with the probiotic *Lacticaseibacillus paracasei* CBAL74 (FM-CBAL74) in preventing pediatric acute gastroenteritis, but the mechanisms of action are still poorly characterized. We evaluated the protective action of FM-CBAL74 in an in vitro model of RV infection in human enterocytes. The number of infected cells together with the relevant aspects of RV infection were assessed: epithelial barrier damage (tight-junction proteins and transepithelial electrical resistance evaluation), and inflammation (reactive oxygen species, pro-inflammatory cytokines IL-6, IL-8 and TNF-α, and mitogen-activated protein kinase pathway activation). Pre-incubation with FM-CBA L74 resulted in an inhibition of epithelial barrier damage and inflammation mediated by mitogen-activated protein kinase pathway activation induced by RV infection. Modulating several protective mechanisms, the postbiotic FM-CBAL74 exerted a preventive action against RV infection. This approach could be a disrupting nutritional strategy against one of the most common killers for the pediatric age.

## Introduction

*Rotavirus* (RV), a segmented double-stranded RNA virus of the *Reoviridae* family, is the most common pathogen identified in children with acute gastroenteritis (AGE) worldwide^1^. It is responsible for up to 50% of severe AGE episodes, > 600.000 deaths and approximately 2.4 million hospitalization annually in young children worldwide^2,3^. To limit RV associated disease burden, in 2009 the World Health Organization (WHO) recommended a global use of RV vaccine in the early pediatric age^4^. A positive impact of RV vaccination has been demonstrated, but the incidence and mortality rate of RV-induced AGE in high- and low-income countries remain to be different from each other. This scenario strongly suggests the necessity of anti-RV strategies to limit the number of deaths deriving from dehydration^5^.

Postbiotics are commonly defined as any factor resulting from the metabolic activity of a probiotic or any released molecule capable of conferring beneficial effects to the host in a direct or indirect way^6^. As postbiotics do not contain living microorganisms, the risks associated with their use in human nutrition are minimized. Postbiotics lack the issue related to the development of antibiotic-resistance gene and the issue of living microorganism exposure to immature immune system and gut barrier, especially in early life^7,8^. From an economic point of view, postbiotics lack the issue related to low temperature storage, facilitating shelf life, packaging and transportation^9^.

Research focused on the biological activities of postbiotics is facilitating their use for preventing and treating infectious diseases^10^. Fermented cow’s milk with *Lacticaseibacillus paracasei* CBAL74 (FM-CBAL74) is among the best characterized and studied postbiotics in the pediatric age^11–16^. Results from two randomized-controlled trials suggested that FM-CBAL74 can prevent AGE in young children^11,12^. A positive regulation of several defense mechanisms, including the modulation of gut barrier, the stimulation of adaptative (secretory immunoglobulin A, sIgA), and of innate immunity (human alpha-defensins 1–3; human beta-defensin 2; cathelicidin LL-37) has been demonstrated in experimental models and in clinical trials^11–14^. In addition, the dietary supplementation with FM-CBAL74 has been associated with a beneficial modulation of gut microbiome structure and function in neonates and young children^15,16^.

Prevention is the key for controlling RV-induced AGE. A well-characterized cellular model of RV infection provided the opportunity to investigate the protective action of FM-CBAL74 against RV-induced AGE. We found that FM-CBAL74 was able to efficiently prevent all main aspects of RV infection with a significant impact on cellular damage and inflammatory response, through the downregulation of mitogen-activated protein (MAP) kinase pathway. Altogether, these data suggest the potential of the postbiotic approach based on the use of FM-CBAL74 in preventing RV-induced AGE. This approach could be a disrupting nutritional strategy against one of the most common killers for the pediatric age.

## Results

### Cellular damage

**Figure 1:**
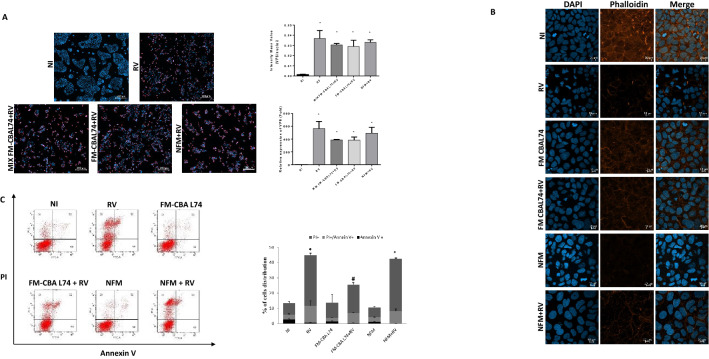
Effects of FM-CBAL74 on cellular damage in *Rotavirus-*infected human enterocytes. **(A)** Caco-2 cells were infected with RV and pretreated with FM-CBAL74 or NFM for 48 h. Cells were fixed and processed for immunofluorescence (IF). VP6 protein was visualized with anti-VP6 and Alexa Fluor-594-conjugated secondary antibody (red) and nuclei were stained with DAPI (blue). Cells were observed through confocal. (Left panel) The infectivity of RV in Caco-2 cells was analyzed by the quantification of VP6 protein by immunofluorescence (IF) staining. The addition of FM-CBAL74 to RV for up to 6 h before infection (MIX FM-CBAL74 + RV) or pre-incubation with FM-CBAL74 (FM-CBAL74 + RV) resulted in no significant modulation of RV infectivity. Representative images of IF are reported in the Figure. Scale bar, 200 µm. (Right panel) The quantification (intensity mean value) of VP6 protein is reported in upper graph. An increase of VP6 mRNA levels, evaluated by RT-PCR, was observed in RV-infected enterocytes in below graph. The incubation of RV with FM-CBAL74 for 6 h before infection (MIX FM-CBAL74 + RV) or pre-incubating the cells with FM-CBAL74 for 48 h before RV infection (FM-CBAL74 + RV) were unable to significantly decrease the VP6 mRNA levels. **(B)** Caco-2 cells were infected with RV and pretreated with FM-CBAL74 or NFM for 48 h. Cells were fixed and processed for immunofluorescence. Actin was visualized using phalloidin-TRITC (red) and nuclei were stained with DAPI (blue). RV-infected enterocytes showed a marked alteration of cytoskeleton structure with a disorganization of F-actin filaments. Disorganized actin in RV-infected cells is shown within yellow squares. Non-infected cells showed a regular distribution of cytoskeleton actin filaments. Pre-incubation with FM-CBAL74 (FM-CBAL74 + RV), but not with NFM (NFM + RV), protected the cells against RV-induced F-actin filaments rearrangements. Representative images of IF are shown in the Figure. Scale bar, 10 µm. **(C)** Caco-2 cells were infected with RV pretreated with FM-CBAL74 or NFM for 48 h. Apoptotic cell rate was assessed by annexin V assay using flow cytometry. An increase in necrotic cells (positive only for propidium, PI) and late apoptotic cells (positive for both PI and Annexin V) confirmed the pro-apoptotic effect induced by RV infection compared to non-infected cells. Pre-incubation with FM-CBAL74 (FM-CBAL74 + RV), but not with NFM (NFM + RV), prevented these effects. Data represent the means with SD of 3 independent experiments, each performed in triplicate. Data were analyzed using the one-way ANOVA test. *p < 0.05 *vs* non-infected cells (NI); ^#^p < 0.05 *vs* RV-infected cells; °p < 0.05 *vs* NFM + RV. *RV* Rotavirus, *FM-CBAL74* fermented milk *L. paracasei* CBAL74, *NFM* not fermented cow milk.

The RV infectivity is commonly demonstrated by the immunofluorescence staining of viral capsid protein VP6 in human enterocytes^17,18^. The RV infection of Caco-2 cells was confirmed by an increase of VP6 protein quantification and mRNA levels (*p* < 0.001) (Fig. 1, panel A). The co-incubation of RV with FM-CBAL74 for 6 h before infection or the pre-incubation of RV with FM-CBAL74 for 48 h before infection were unable to limit the number of infected enterocytes (Fig. 1, panel A).

RV infection induces alterations of the cytoskeleton through ERK pathway activation via phosphorylating actin cross-linking/bundling proteins^19^. This mechanism induces a rearrangement of F-actin filaments^20^. As showed in Fig. 1, panel B, non-infected cells showed a regular distribution of cytoskeleton actin filaments. In contrast, RV-infected enterocytes showed a marked alteration of cytoskeleton structure with a disorganization of F-actin filaments. Pre-incubation with FM-CBAL74, but not with non-fermented cow’s milk (NFM), protected the cells against RV-induced rearrangements of F-actin filaments (Fig. 1, panel B).

RV infection induces apoptosis in human enterocytes^21,22^. An increase in necrotic cells (positive only for propidium, PI) and late apoptotic cells (positive for both PI and Annexin V) confirmed the pro-apoptotic effect induced by RV infection (*p* < 0.05) (Fig. 1, panel C). Pre-incubation with FM-CBAL74, but not with NFM, prevented these effects (*p* < 0.05) (Fig. 1, panel C).

These results suggested that, while FM-CBAL74 is unable to prevent RV infection in human enterocytes, it can limit the subsequent cytoskeleton alterations and apoptosis.

### MAP kinases pathway activation

**Figure 2:**
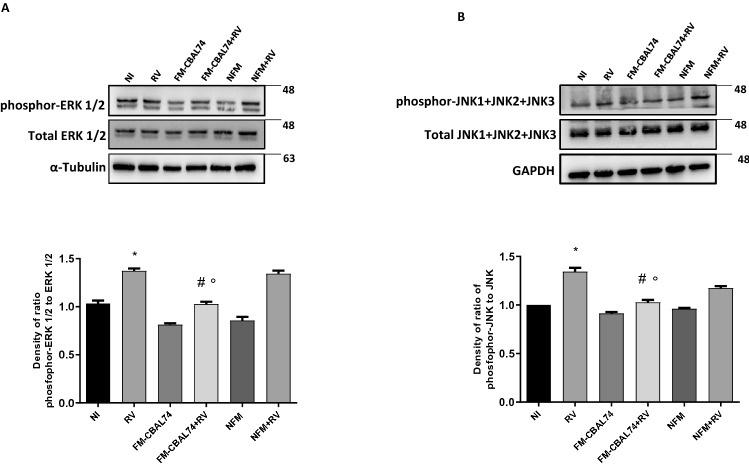
Effects of FM-CBAL74 on MAP kinases pathway activation in *Rotavirus*-infected human enterocytes. **(A,B)** Caco-2 cells were infected with RV and pretreated with FM-CBAL74 or NFM for 48 h. Western blot assay of phospho-ERK/total ERK **(A)** and phospho-JNK/total JNK **(B)** was performed on protein extracts from Caco-2 cells. RV infection induced MAP kinases ERK, and JNK expression significantly increased compared with non-infected cells. Pre-incubation with FM-CBAL74 (FM-CBAL74 + RV), but not with NFM (NFM + RV), down-regulated this pro-inflammatory pathway in Caco-2 cells. The amounts of these proteins and of β-actin were measured by Western blot. The histogram below shows optical density of the proteins, obtained with Image Lab software. Relative quantification of proteins was normalized versus β-actin protein and was calculated using the ratio between phosphorylated and total proteins. The figure showed representative image of three experiments qualitatively similar. In Supplementary Information 1 is provided the full length WB gels of these proteins.Data represent the means with SD of 3 independent experiments, each performed in triplicateData were analyzed using the one-way ANOVA test. *p < 0.05 vs non-infected cells (NI); ^#^p < 0.05 vs RV-infected cells; °p < 0.05 vs NFM + RV. *RV* Rotavirus, *FM-CBAL74* fermented milk *L. paracasei* CBAL74, *NFM* not fermented cow milk.

The activation of the extracellular signal-regulated kinase (ERK) and c-Jun-N-terminal kinase (JNK), has been described in RNA viruses’ infection^23^. The phosphorylation status of these kinases was investigated. Pre-treatment with FM-CBAL74 prevented the RV-induced phosphorylation ratio increase of ERK and JNK (*p* < 0.001) (Fig. 2, panels A,B).

These results provided evidence on ERK/JNK pathway involvement in the protective action of FM-CBAL74 against RV infection.

### Intestinal permeability

To investigate the protective action of FM-CBAL74 against gut barrier alteration induced by RV infection, we evaluated the transepithelial electric resistance (TEER), the expression of the tight junction (TJ) proteins, occludin and zonula occludens -1 (ZO-1), and of the cell adhesion molecule E-cadherin in Caco-2 cells monolayer.

**Figure 3:**
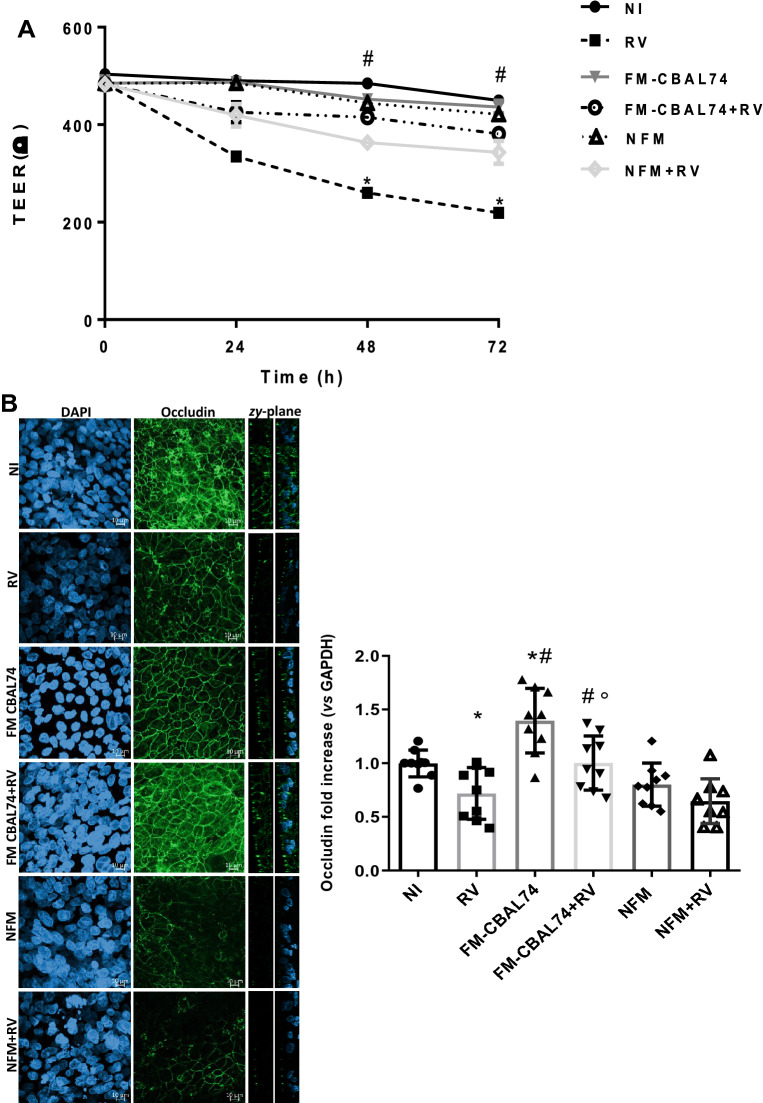

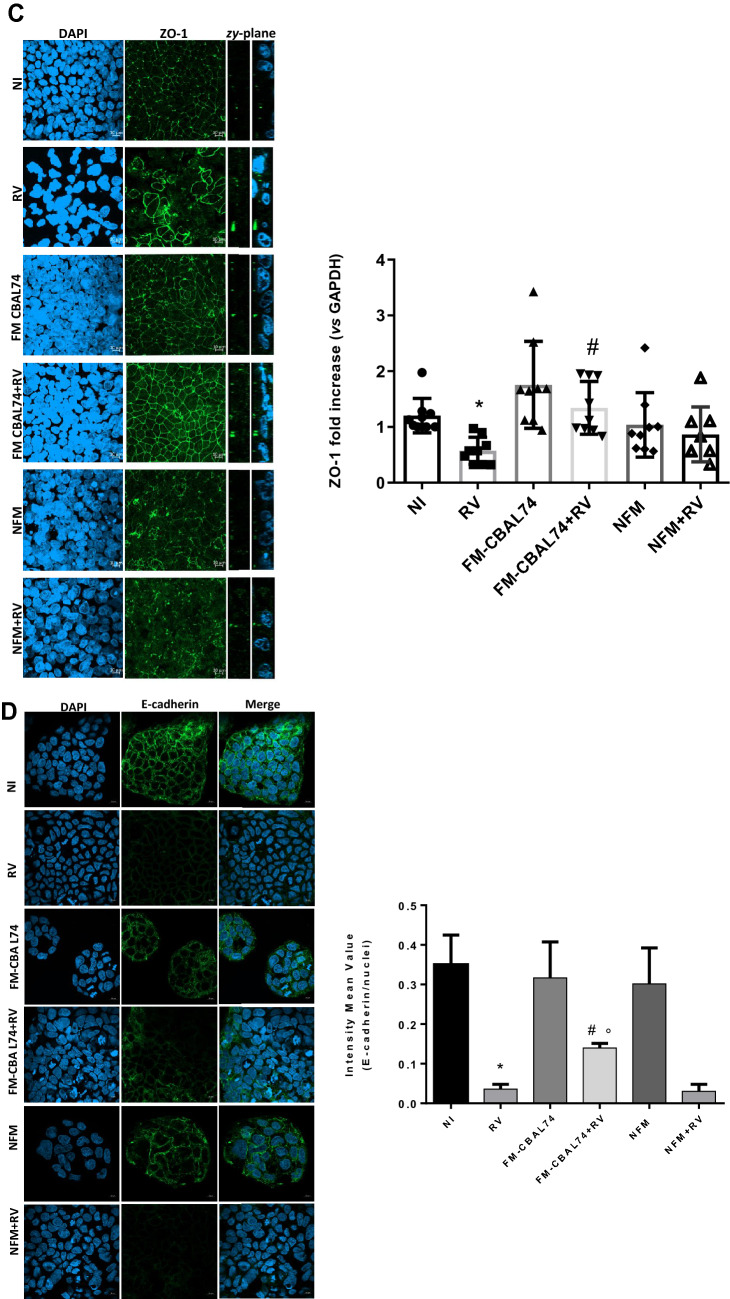
Effects of FM-CBAL74 on gut permeability in *Rotavirus*-infected human enterocytes. **(A)** Caco-2 cells were infected with RV and pretreated with FM-CBAL74 or NFM for 48 h. RV infection affected intestinal epithelial permeability, as demonstrated by TEER measurement up to 72 h of incubation. Pre-incubation with FM-CBAL74 (FM-CBAL74 + RV) significantly inhibited this effect. The TEER values were measured as follows: TEER = (measured resistance value − blank value) × single cell layer surface area (cm^2^). **(B,C)** Caco-2 cells were infected with RV and pretreated with FM-CBAL74 or NFM for 48 h. Cells were processed for mRNA analysis and fixed for immunofluorescence (IF). (Left (**B,C**)). Occludin and ZO-1 were visualized with anti-occludin and Alexa Fluor-488-conjugated secondary antibody (green) and with anti-ZO-1 and Alexa Fluor-488 -conjugated secondary antibody (green) and nuclei were stained with DAPI (blue). Cells were observed through confocal microscope in the zy-plane. RV infection elicited a redistribution of occludin and ZO-1 proteins in Caco-2 cells and reduced their expression (Right (**B,C**)). Pretreatment of RV-infected cells with FM-CBAL74 (FM-CBAL74 + RV) prevented the redistribution of occludin and ZO-1 proteins (Left (**B,C**)) and their mRNA reduction in Caco-2 cells monolayer (Right (**B,C**)). NFM was unable to modulate the expression of occludin and ZO-1 in RV-infected cells. Representative images of IF are shown. Scale bar, 10 µm. **(D)** Caco-2 cells were infected with RV and pretreated with FM-CBAL74 or NFM for 48 h. Cells were fixed and processed for IF. E-cadherin was was visualized with anti-E-cadherin and Alexa Fluor-488-conjugated secondary antibody (green) and nuclei were stained with DAPI (blue). RV infection reduced E-cadherin expression compared to non-infected cells. Pre-incubation of RV-infected cells with FM-CBAL74 (FM-CBAL74 + RV) significantly prevented the reduction of E-cadherin expression in Caco-2 cells monolayer. NFM was unable to modulate the expression of E-cadherin in RV-infected cells. Representative images of IF (left panel) and Intensity mean value of E-cadherin (right panel) are shown. Scale bar, 10 µm. Data represent the means with SD of 3 independent experiments, each performed in triplicate. Data were analyzed using the one-way ANOVA test. *p < 0.05 vs non-infected cells (NI); ^#^p < 0.05 vs RV-infected cells; °p < 0.05 vs NFM + RV. *RV* Rotavirus, *FM-CBAL74* fermented milk *L. paracasei* CBAL74, *NFM* not fermented cow milk.

RV infection determined a significant decrease of transepithelial resistance (TEER) in human enterocytes (*p* < 0.05). This event was associated with an alteration of TJ proteins structure, as demonstrated by redistribution of occludin, ZO-1 and E-cadherin (Fig. 3). The pre-treatment with FM-CBAL74 prevented RV-induced TEER decrease *(p* < 0.05) (Fig. 3, panel A). The redistribution of occludin and ZO-1 has been associated with TJ proteins alteration and barrier dysfunction in gut epithelium^24^. RV infection in Caco-2 cells caused an alteration of TJ proteins, as demonstrated by occludin and ZO-1 redistribution (Fig. 3, panels B,C) and by reduction of their expression (*p* < 0.05) (Fig. 3, panels B,C). Pre-treatment with FM-CBAL74, but not with NFM, protected Caco-2 cells from occludin and ZO-1 redistribution (Fig. 3, panel B,C) and enhanced their expression (*p* < 0.05) (Fig. 3 panels B,C), suggesting a protective effect against gut barrier dysfunction. Furthermore, we found that E-cadherin protein appeared significantly reduced in RV-infected cells (*p* < 0.001) (Fig. 3, panel D). Again, pre-treatment with FM-CBAL74, but not with NFM, significantly prevented the reduction of E-cadherin expression caused by RV infection (*p* < 0.05) (Fig. 3, panel D).

These results showed that FM-CBAL74 was able to prevent RV induced alteration of gut barrier integrity. In Supplementary Information 3, we provide a 3D video reconstruction from Z-stack acquisition of Occludin. 

### Inflammatory response

Oxidative stress and inflammation are closely related pathophysiological events in infectious diseases^25^. To further investigate the protective action of FM-CBAL74 on inflammatory response induced by RV infection, we investigated oxidative stress (ROS production) and pro-inflammatory cytokines (IL-6, IL-8 and TNF-α) response in human enterocytes.

**Figure 4:**
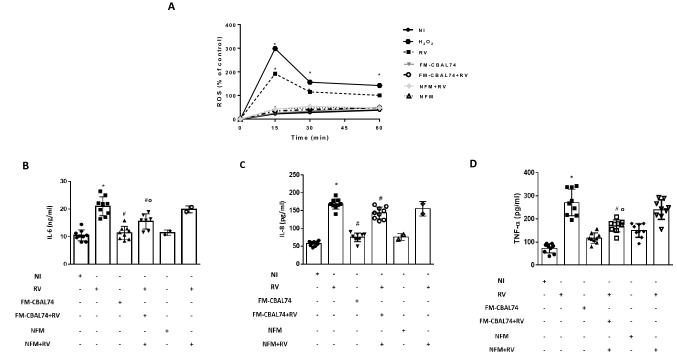
Effects of FM-CBAL74 on ROS and pro-inflammatory cytokines production in *Rotavirus*-infected human enterocytes. **(A)** Caco-2 cells were infected with RV and pretreated with FM-CBAL74 or NFM for 48 h. RV induced a significant increase in ROS production in a time-dependent manner. Pre-incubation with FM-CBA L74 (FM-CBAL74 + RV), but not with NFM (NFM + RV), significantly inhibited the RV-induced increase in ROS. H_2_O_2_ served as a positive control. **(B–D)** Caco-2 cells were infected with RV and pretreated with FM-CBAL74 or NFM for 48 h. The supernatants were collected for ELISA assays. RV elicited a significant increase in IL-6 **(B)**, IL-8 **(C)** and TNF-α **(D)** production. Pre-incubation with FM-CBAL74 (FM-CBAL74 + RV), but not with NFM (NFM + RV), significantly inhibited the RV-induced increase in IL-6, IL-8 and TNF-α production in Caco-2 cells. Data represent the means with SD of 3 independent experiments, each performed in triplicate. Data were analyzed using the one-way ANOVA test. *p < 0.05 *vs* non-infected cells (NI); ^#^p < 0.05 *vs* RV-infected cells; °p < 0.05 *vs* NFM + RV. *RV* Rotavirus, *FM-CBAL74* fermented milk *L. paracasei* CBAL74, *NFM* not fermented cow milk.

As shown in Fig. 4, panel A, RV significantly increased ROS production. The pretreatment with FM-CBAL74, but not with NFM, inhibited the RV-induced ROS increase (*p* < 0.05) (Fig. 4, panel A). In parallel, FM-CBAL74, but not NFM, inhibited IL-6, IL-8 and TNF-α production induced by RV infection in human enterocytes (*p* < 0.05) (Fig. 4, panels B–D).

Altogether these data suggested that FM-CBAL74 was able to inhibit ROS production and IL-8, IL-6 and TNF-α cytokines release induced by RV infection in human enterocytes.

## Discussion

Emerging evidence suggest the potential of the postbiotic approach for the prevention of pediatric infectious diseases^6–16,26,27^. Unfortunately, discrepancies in clinical results evaluating different postbiotic products, and the poor definition of the mechanisms of action elicited against specific pathogens are blunting the wide use of this approach in clinical practice.

Rotavirus is the most common agent of AGE in the pediatric age^2,3^. Our study aimed to explore the anti-RV effect of a specific postbiotic deriving from the fermentation of cow’s milk with the probiotic *L. paracasei* CBAL74 with a demonstrated clinical efficacy against pediatric AGE^11,12^. We found that this postbiotic positively modulates a range of non-immune and immune defense mechanisms against RV infection in human enterocytes.

Even though this postbiotic was unable to significantly reduce the number of infected cells, a protective action against gut barrier damage, characterized by cytoskeleton alterations and apoptosis, was observed. The effect involved the modulation of a pivotal regulator of stress-induced cell damage, the ERK/JNK kinase pathway^28–32^. ERK/JNK pathway is activated by different viruses, including influenza A, herpes simplex virus 1, hepatitis C virus, and RV^23,32–35^. Components of RV outer capsid proteins, such as VP4-VP8 and VP4-VP5 domains, VP7, and the non-structural proteins NSP1 activate PI3K/Akt and ERK/JNK pathways^23,36^.

According to previous evidence^37,38^, our data demonstrated that RV infection stimulates ROS production in human enterocytes. The increased ROS production induces the release of pro-inflammatory cytokines (IL-8, IL-6, TNF-α) through the activation of MAP kinase pathway^25,39–42^. We demonstrated that FM-CBAL74 was able to prevent RV-induced ROS, IL-8, IL-6 and TNF-α production, suggesting an inhibition of the inflammatory “cytokine storm” which in turn is responsible for the severity of signs and symptoms commonly observed in children infected by this microorganism^2^.

A major strength of our study is the evaluation of all crucial steps of RV infection in a well validated experimental model^43–46^. The major limitation resides in the lack of evidence on which specific FM-CBAL74 component could be responsible for the protective effects. Several components of this postbiotic could be involved, including lipoteichoic acid, peptidoglycans, bacteriocins, nucleotides and peptides. It has been demonstrated that peptides deriving from fermentation of cow’s milk proteins could act as modulators of non-immune and immune gastrointestinal defense mechanisms^47–49^. We have previously demonstrated that this postbiotic could exert a positive modulation of gut microbiome in the pediatric age associated with increased production of secretory IgA (sIgA) and butyrate^14–16^. Considering the relevant role exerted by these molecules in protecting against infections and in modulating gut barrier integrity and inflammation, it is possible to hypothesized that the protective actions reported in this study could be further reinforced by the modulation of sIgA and gut microbiome^10^. Future studies on the efficacy and mechanisms of action of FM-CBAL74 using more complex systems, such as human biopsies and/or organoids exposed to different gastrointestinal pathogens, are advocated to further explore the potential of this approach. Another limitation is related to the fact that we explored the protective effects of FM-CBAL74 against only the main agent of pediatric AGE. Similar protective action against *Salmonella typhimurium* has been reported by others^13^. Future studies are advocated to better elucidate the protective effect elicited by this postbiotic against other microorganisms responsible for pediatric AGE.

In conclusion, we provided evidence on the protective action elicited by FM-CBAL74 against the most common agent of pediatric AGE. A range of intracellular mechanisms has been highlighted. These mechanisms could act in parallel with other beneficial actions on gut microbiome structure and function, and on innate and adaptive immunity that has been already demonstrated in children receiving this postbiotic^11–16^. Altogether, these data could pave the way to innovative nutritional strategies against one of the most common killers for the pediatric age, responsible for > 600,000 deaths yearly worldwide^3^.

## Methods

### Caco-2 cell line

For all experiments, we used Caco-2 cell line (American Type Culture Collection, Middlesex, UK; accession number: HTB-37). These cells show the typical features of small intestine human enterocytes^50^ and they are commonly adopted for experiments exploring the effects of pathogens, drugs, probiotics, and nutrients^51^. The Caco-2 cells were cultured in high glucose Dulbecco’s modified Eagle medium (DMEM; Gibco, Berlin, Germany) with 10% fetal bovine serum (Sigma-Aldrich; St. Louis, Missouri, USA), 1% on-essential aminoacids (Sigma-Aldrich; St. Louis, Missouri, USA), 1% (v/v) antibiotics (10.000 U/mL penicillin and 10 mg/mL streptomycin) (Euroclone Spa; MI, Italy), and 4 mM l-glutamine (Sigma-Aldrich; St. Louis, Missouri, USA). Caco-2 cells were grown in an incubator at 37 °C and 5% CO_2_. The culture medium was changed every 2 days. All experiments were carried out three times in triplicate.

### Rotavirus strain

The simian *Rotavirus* strain SA11 was provided by ATCC (accession number: VR-1565™). Virus stock was grown in MA104 cells (ATCC; accession number: CRL-2378.1™), which were maintained in Medium 199 (Lonza, Basel, Switzerland) without serum and supplemented with 20 µg/mL of trypsin from porcine pancreas type IX (Sigma-Aldrich, St. Louis, Missouri, USA) for 96 h. Subsequently, the cells were lysed by freezing and thawing to achieve virus release. Extracted virus was titrated by focus forming assay (FFA) and expressed in focus-forming units (ffu) per cell, as previously described^22^. The SA11 strain is well characterized and is able to replicate to high titers in Caco-2 cells compared to other *Rotavirus* strains^43^.

### Study products

Study products were provided by Kraft-Heinz Italia, SpA, Latina, Italy an affiliate of Kraft-Heinz Company, co-headquartered in Pittsburgh, PA and Chicago, IL, USA). The preparation of fermented cow milk with *L. paracasei* CBA L74 (International Depository Accession Number LMG P-24778) was performed as previously described^14^. Briefly, the fermentation was started in the presence of 10^6^ bacteria, reaching 5.9 × 10^9^ colony-forming units/g after 15 h incubation at 37 °C. After heating at 85 °C for 20 s, in view of inactivating the live bacteria, the formula is spray dried. The final fermented cow milk powder contained only bacterial bodies and fermentation products and no living microorganisms. The control (non-fermented cow milk, NFM) consisted of skimmed milk powder with the same basal nutrients’ composition of fermented milk powder (grams per 100 g): proteins, 35; lipids, 1; carbohydrates, 54.

### Rotavirus activation and infection protocol

*Rotavirus* strain SA11 activation was performed as previously described^43^. Briefly, the virus was activated with 20 µg/mL trypsin from porcine for 1 h at 37 °C. The viral suspension was added to the apical side of cell monolayers. After 60 min, the cells were washed and incubated in FBS-free medium for the indicated time periods after infection. We adopted two infection protocols: (i) in the first infection protocol, FM-CBAL74 and NFM at the dose of 11.5 mg/mL were co-incubated with RV (25 ffu/cell), previously activated, in a sterile tube for 1 h and then, used to stimulate the cells for 48 h; (ii) in the second infection protocol, Caco-2 cells were pre-treated with 11.5 mg/mL of FM-CBAL74 and NFM for 48 h at 37 °C before infection. The dose of 11.5 mg/mL was established in dose–response experiments confirming previous results obtained by our group^14^. Then, the viral suspension (25 ffu/cell) was added to the Caco-2 monolayer for 1 h. After inoculation, cells were washed twice to remove free viruses and maintained with serum-free medium after infection.

### Quantification of Rotavirus infection

To quantify RV infection, 2.5 × 10^5^ undifferentiated Caco-2 cells were washed and fixed with ice-cold methanol (Carlo Erba Reagents; Milan, Italy) for 10 min at room temperature. Then, the cells were washed twice with phosphate-buffered saline (PBS) (Gibco, Berlin, Germany) and permeabilized with Triton X-100 (PanReac AppliChem) for 10 min. After washing, the cells were blocked for 1 h using 1% bovine serum albumin (BSA; PanReac AppliChem) in PBS/Tween 20 (PanReac AppliChem) and then incubated overnight at 4 °C with specific primary antibody for intermediate capsid protein VP6 of RV (1:100; Abcam, ab181695). The secondary antibody, an anti-mouse (1:500; Alexa Fluor 594, Invitrogen, MA, USA), was incubated in blocking solution for 1 h. Nuclei were stained with 4′,6-Diamidino-2-phenylindole dihydrochloride (DAPI) (Invitrogen). Finally, cells were mounted with antifading Mowiol (Sigma-Aldrich; St. Louis, Missouri, USA) and analyzed using an inverted fluorescence microscope.

### Apoptosis (annexin V assay) by FACS analysis

To analyze cell apoptosis rate, 2.5 × 10^5^ undifferentiated Caco-2 cells were plate in 6-well plates and Annexin V Apopstosis Detection Kit APC was used (eBioscience, San Diego, CA, USA) according to the manufacturer’s protocol, as previously described^17^. After 48 h of treatment, the cells were washed and incubated with 1× Annexin V binding buffer, then 5 × 10^5^ cells were stained with Annexin V-fluorescein isothiocyanate (FITC) for 10 min at room temperature in the dark. Before reading with a BD FACS Calibur flow cytometer (Becton Dickinson, Franklin Lakes, NJ, USA), propidium iodide (PI) 5 µg/mL was added.

### Transepithelial electrical resistance measurement

To evaluate the monolayer integrity by transepithelial electrical resistance (TEER), 2 × 10^6^ Caco-2 cells per well were seeded on polycarbonate 6-well Transwell^®^ membranes (Corning, Life Science, Kennebunk, USA). After 15 days post-confluence, the TEER of monolayer was measured every 24 h for a total of 72 h, using an epithelial Volt-Ohm Meter (Millicel-ERS-2, Millipore, Billerica, MA, USA). Transepithelial resistance was measured at 24, 48 and 72 h after RV infection. The measured resistance value was multiplied by the area of the filter to obtain an absolute value of TEER, expressed as Ω cm^2^ and the TEER values were measured as follows: TEER = (measured resistance value − blank value) × single cell layer surface area (cm^2^).

### Reactive oxygen species production

Reactive oxygen species (ROS) production was measured by 7′-dichlorofluorescein diacetate (DCFH-DA) (Sigma-Aldrich) spectrofluorometry on differentiated Caco-2 cells, as previously described^43^. Briefly, after stimulation, DCFH-DA (20 µM) was added for 30 min at 37 °C in the dark. After twice washes in PBS, intracellular ROS production was measured in a fluorometer (SFM 25, Kontron Instruments; Japan). As a positive control, hydrogen peroxide (H_2_O_2_) (Sigma-Aldrich) was used at concentrations of 10 mM for 15, 30 and 60 min.

### Quantitative real-time PCR

Total RNA was isolated from cells with TRizol reagent (Sigma-Aldrich) and quantified using a NanoDrop Spectrophotometer and purity was verified by A260/280 and A260/230 absorbance ratios. The integrity of the RNA was checked using gel electrophoresis. RNA (500 ng) was reverse transcribed in cDNA with a High-Capacity RNA-to-cDNA™ Kit (Applied Biosystems; Vilnius, Lithuania) according to the manufacturer’s instructions. Complementary DNA (cDNA) was stored at − 80 °C until use. Quantitative real-time PCR (qRT-PCR) analysis was performed using Taqman Gene Expression Master Mix (Applied Biosystems, Grand Island, NY, USA) to evaluate the effect of intestinal exposure to milk products and *Rotavirus* SA11 on the gene expression of TJ occludin and ZO-1 (Hs00170162_m1 and Hs01551871_m1, respectively). The TaqMan probes for these genes were inventoried and tested by Applied Biosystems manufacturing facility (QC).

RV-VP6 expression was evaluated using a SYBR green Master Mix (Applied Biosystems, Grand Island, NY, USA). The primers used were: VP6 F 5′-GCACAGCCATTCGAACATCATGC-3′; VP6 R 5′-TGCATCGGCGAGTACAGACTC-3′. Amplification conditions were initial steps at 50 °C for 2 min and 95 °C for 10 min, followed by 40 cycles of 95 °C for 15 s and 60 °C for 1 min in a Light Cycler 7900HT (Applied Biosystems). The expression of each gene was normalized to that of Glyceraldeide-3-Phosphate Dehydrogenase (TaqMan assay: GAPDH; Hs02786624_g1, primers for SYBR Green assay: GAPDH F 5′-AATCCCATCACCATCTTCCAG-3′; GAPDH R 5′-AATGAGCCCCAGCCTTC-3′) to normalize a relative transcript level. Relative gene expression was calculated by the 2^−ΔΔCT^ method: (ΔΔCT = ΔCT_sample_ − ΔCT_control_). Each sample was analyzed in triplicate.

### Analysis of pro-inflammatory cytokines production

The concentrations of IL-8, IL-6 and TNF-α were analyzed in cell supernatants collected after treatment and stored at − 80 °C. The three cytokines production was measured by ELISA using commercially available kits (Abcam, Cambridge, USA) according to the manufacturer’s instructions, and results expressed in pg/mL. The detection limits of IL-8, IL-6 and TNF-α were 1.8 pg/mL, 2 pg/mL and 30 pg/mL, respectively.

### Western blot analysis

Western blotting analysis was carried out following proteins cell extraction by RIPA buffer (50 mM Tris–Hcl, pH 7.6, 150 mM NaCl, 1 mM MgCl_2_, 1% NP-40) supplemented with a protease and phosphatase inhibitor cocktail. Protein concentrations were estimated using BioRad protein assay dye reagent and BSA (PanReac AppliChem) as standard. Proteins (30 µg) were separated by SDS–Polyacrylamide gel electrophoresis and subsequently transferred onto Polyvinylidene fluoride (PVDF) membranes (Immobilon^R^-Transfer Membrane, Tullagreen, Carrigtwohill, Co). Nonspecific protein binding was blocked with a solution containing 5% nonfat dry milk (PanReac AppliChem) and 0.2% Tween20/PBS for 1 h at room temperature. Specific primary antibodies for p-ERK1/2 (Thr202/Tyr204) (1:1000; Abcam, ab32538), total ERK1/2 (1:1000; Abcam, ab17942), p-JNK1 + JNK2 + JNK3 (T183 + T183 + T221) (1:1000; Abcam, ab124956), total JNK1 + JNK2 + JNK3 (1:1000; Abcam, ab179461), α -Tubulin (1:5000; Sigma-Aldrich, T6074) and GAPDH (1:5000; Sigma-Aldrich, G8795) were incubated overnight at 4 °C sequentially the peroxidase-linked (HRP) conjugated anti-rabbit IgG (1:2000; Abcam, ab205718) or anti-mouse IgG (1:5000; ImmunoReagents, GtxMu-003-DHRPX) and enhanced Chemiluminescence solution (ECL Wester Antares; Cyanagen) were used for visualizing protein expression. The relative band intensity of each protein was obtained by the normalization to the band intensity of GAPDH, and α-tubulin loading control, using Image Lab Software (Biorad, Hercules, CA, USA).

### Immunofluorescence and confocal microscopy

For actin cytoskeleton detection, 2.5 × 10^5^ undifferentiated Caco-2 cells were washed and fixed with 4% paraformaldehyde (PFA) (Carlo Erba Reagents) for 10 min at room temperature. Autofluorescence due to free aldehyde groups from PFA treatment were blocked with 50 mM Ammonium Chloride (Sigma-Aldrich) in PBS for 10 min at room temperature. Cover slips were washed twice with PBS, then cells were permeabilized with Triton X-100 (PanReac AppliChem) in PBS for 10 min. After washing, the cells were blocked for 1 h using 1% BSA in PBS/Tween 20 and then incubated for 1 h at room temperature with phalloidin-TRITC (Sigma-Aldrich). At the end, the cells were washed with PBS and mounted with antifading Mowiol. Glass slides were allowed to cure overnight, in the dark.

To investigate TJ and adherent junction proteins, Caco-2 cells, after 15 days post-confluence, were washed and fixed, respectively, with ice-cold methanol and 4% PFA for 10 min at room temperature. Then, the cells were washed twice with PBS and permeabilized with Triton X-100 in PBS for 10 min. After washing, the cells were blocked for 1 h using 1% BSA in PBS/Tween 20 and then incubated overnight at 4 °C with specific primary antibody for occludin (1:100; Abcam, ab31721), ZO-1 (1:100; Abcam, ab96587) and E-cadherin (1:100, BD, #610181). Coverslips were washed with PBS and incubated with horseradish peroxidase (HRP)-conjugated goat anti-rabbit IgG secondary antibody (1:200; Alexa Fluor 488, Invitrogen) or anti-mouse (1:400; Alexa Fluor 488, Invitrogen) for 1 h at RT. Nuclei were stained with DAPI. Finally, cells were mounted in Mowiol. Glass slides were allowed to cure overnight, in the dark. Cells were observed with 63× objective on a Zeiss LSM980 confocal system equipped with an ESID detector and controlled by a Zen blue software (Zeiss; Jena, Germany). Fluorescence images presented are representative of cells imaged in at least three independent experiments.

### Quantification and statistical analysis

The Kolmogorov–Smirnov test was used to determine whether variables were normally distributed. Descriptive statistics were reported as means and standard deviations (SDs) for continuous variables. Data were analyzed using the one-way ANOVA test. The level of significance for all statistical tests was two-sided, p < 0.05. All data were collected in a dedicated database and analyzed by a statistician using GraphPad Prism 7 (La Jolla, CA, USA). The Kolmogorov–Smirnov test was used to determine whether the variables were normally distributed.

## Supplementary Information

Supplementary Information 1.

Supplementary Video legends.

Supplementary Videos.

## Data Availability

All source data are available upon request (Prof. Roberto Berni Canani; berni@unina.it).
