# Supplementary material for: Protective effects of the postbiotic deriving from cow’s milk fermentation with L. paracasei CBA L74 against Rotavirus infection in human enterocytes
Source: Sci Rep. 2022 Apr 15;12:6268. doi: 10.1038/s41598-022-10083-5 (PMC9012738; doi:10.1038/s41598-022-10083-5)

**Supplementary information**

**Protective effects of the postbiotic derived from cow’s milk fermentation with *L.paracasei* CBA L74 against *Rotavirus* infection in human enterocytes**

Cristina Bruno^ǂ^, Lorella Paparo^ǂ^, Laura Pisapia, Alessia Romano, Maddalena Cortese, Erika Punzo and Roberto Berni Canani

**Full-length gel of Phosphor-ERK, Total-ERK,** **Phosphor-JNK, Total-JNK**

Phosphor-ERK **(A),** Total-ERK **(B)** and α-tubulin were blotted on the same gel (PW: 44/42, 44/42 and 52 kDa, respectively). Phosphor-JNK **(D)**, Total-JNK **(E)** and GAPDH **(F)** were blotted on the same gel (PW: 46,46 and 37 kDa, respectively). At left side of the image was represented the molecular weight markers (#G266, Opti-Protein XL Marker, Applied Biological Materials Inc. Richmond, Canada).

Starting at left side of the gel:

Lane 1: NI

Lane 2: RV (25 ffu/cell)

Lane 3: FM-CBAL74

Lane 4: FM-CBAL74+RV

Lane 5: NFM

Lane 6: NFM+RV

*NI, non-infected cells; RV, Rotavirus; FM-CBAL74, fermented milk Lactobacillus paracasei CBA L74; NFM, not fermented milk.*

**(A)**


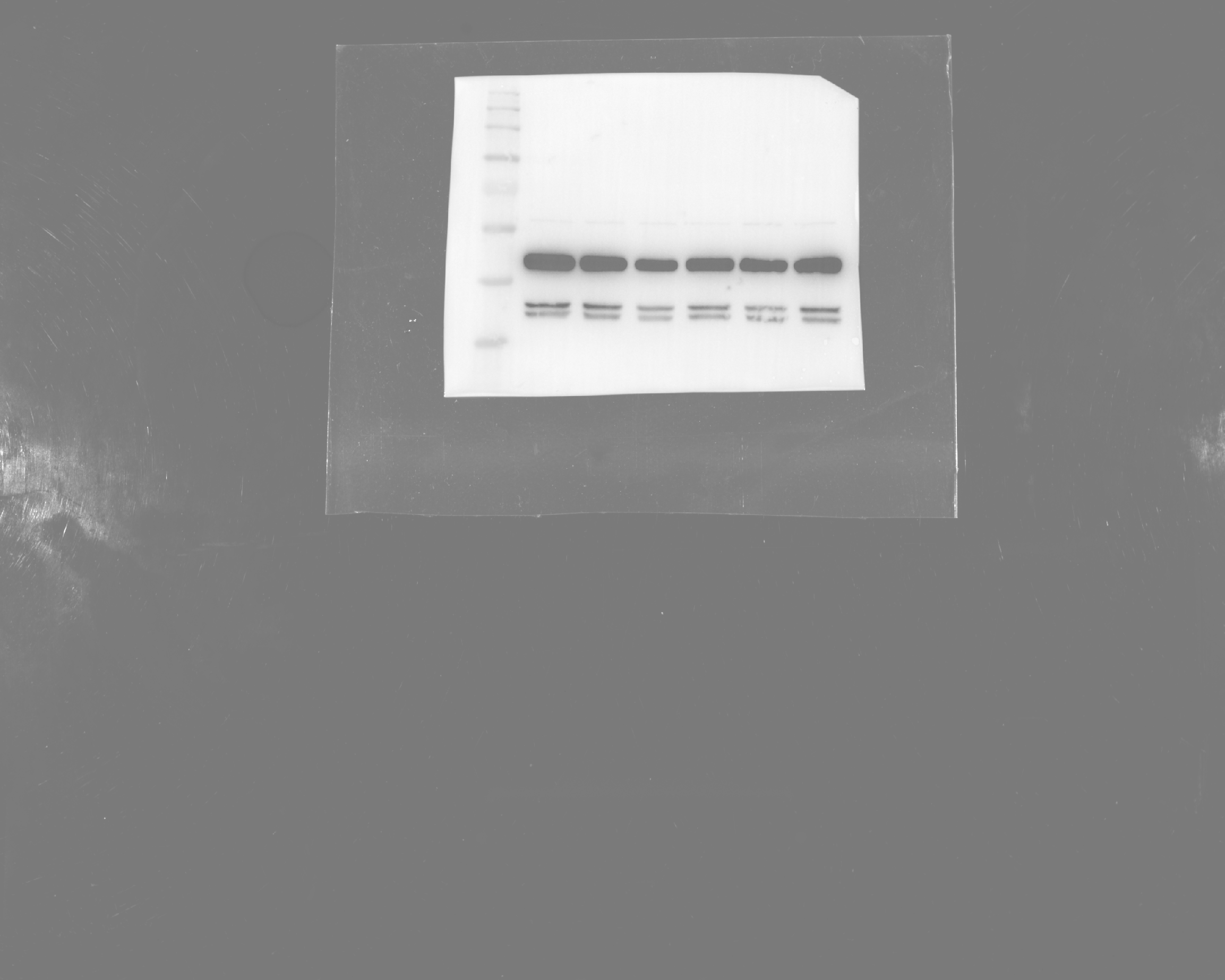


**phosphor-ERK 1/2**

**44/42 kDa**

**(B)**

**
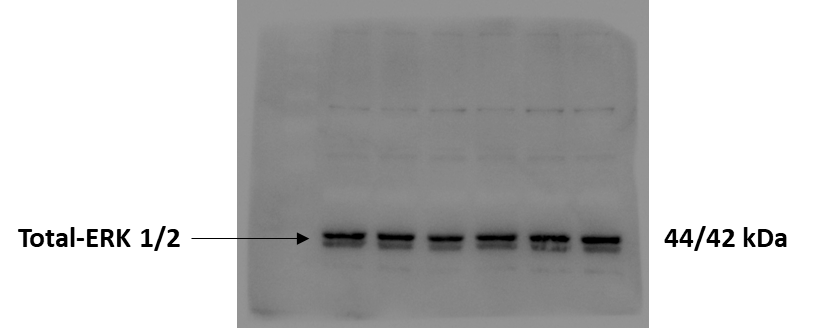
**

**(C)**

**
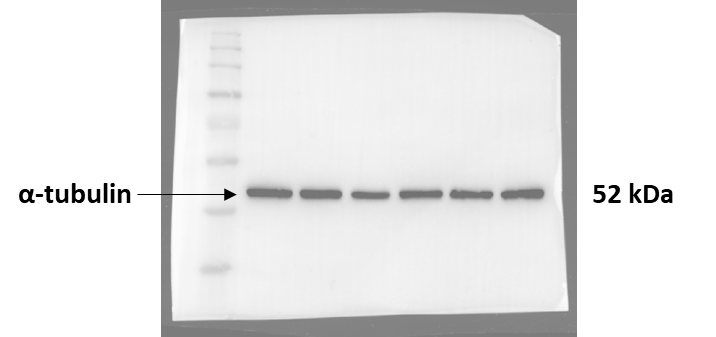
**

**(D**

**
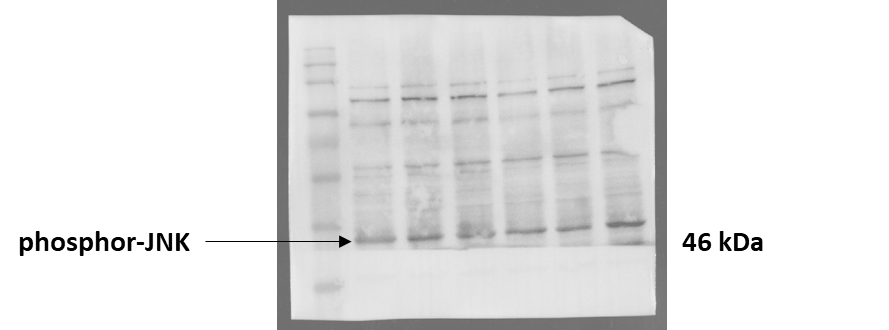
**

**(E)**


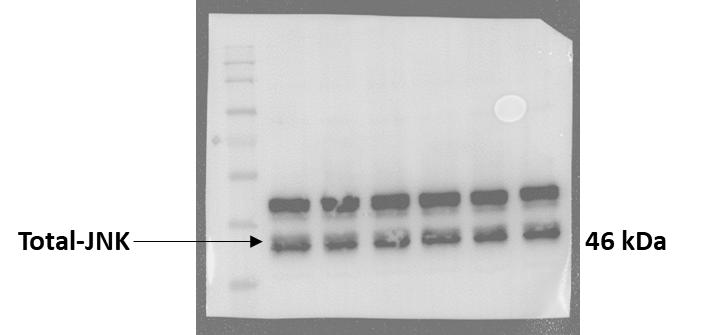


**(F)**


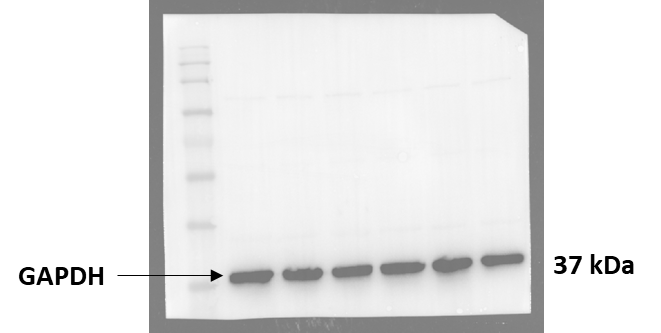

Supplement: Supplementary file 1 — Supplementary Information 1. [file 41598_2022_10083_MOESM1_ESM.doc]
