# Supplementary material for: Protective effects of the postbiotic deriving from cow’s milk fermentation with L. paracasei CBA L74 against Rotavirus infection in human enterocytes
Source: Sci Rep. 2022 Apr 15;12:6268. doi: 10.1038/s41598-022-10083-5 (PMC9012738; doi:10.1038/s41598-022-10083-5)
Supplement: Supplementary file 2 — Supplementary Video legends. [file 41598_2022_10083_MOESM2_ESM.docx]

**Supplementary files**

**Protective effects of the postbiotic derived from cow’s milk fermentation with *L.paracasei* CBA L74 against *Rotavirus* infection in human enterocytes**

Cristina Bruno^ǂ^, Lorella Paparo^ǂ^, Laura Pisapia, Alessia Romano, Maddalena Cortese, Erika Punzo and Roberto Berni Canani

**Video#1.** **3D-reconstruction from Z-stack acquisition of occludin in non-infected human enterocytes.** Occludin was visualized using Alexa 488 (green) and nuclei were stained with DAPI (blue). Cells were observed through confocal microscope.

**Video#2. 3D-reconstruction from Z-stack acquisition of occludin in** ***Rotavirus*-infected human enterocytes.** Occludin was visualized using Alexa 488 (green) and nuclei were stained with DAPI (blue). Cells were observed through confocal microscope. RV infection elicited a significant redistribution of occludin in Caco-2 cells.

**Video#3. 3D-reconstruction from Z-stack acquisition of occludin in human enterocytes treated with FM-CBAL-74.** Occludin was visualized using Alexa 488 (green) and nuclei were stained with DAPI (blue). Cells were observed through confocal microscope. The treatment with FM-CBAL74 not affected occludin redistribution.

**Video#4. 3D-reconstruction from Z-stack acquisition of occludin in *Rotavirus*-infected human human enterocytes pretreated with FM-CBAL-74 (FM-CBAL74+RV).** Occludin was visualized using Alexa 488 (green) and nuclei were stained with DAPI (blue). Cells were observed through confocal microscope**.** Pretreatment of RV-infected cells with FM-CBAL74 significantly prevented the redistribution of occludin in Caco-2 cells monolayer.

**Video#5. 3D-reconstruction from Z-stack acquisition of occludin in human enterocytes treated with NFM.** Occludin was visualized using Alexa 488 (green) and nuclei were stained with DAPI (blue). Cells were observed through confocal microscope. NFM elicited a redistribution od occluding in Caco-2 cells.

**Video#6. 3D-reconstruction from Z-stack acquisition of occludin in *Rotavirus*-infected human human enterocytes pretreated with NFM (NFM+RV).** Occludin was visualized using Alexa 488 (green) and nuclei were stained with DAPI (blue). Cells were observed through confocal microscope. NFM (NFM+RV) was unable to modulate the occludin redistribution observed in RV-infected cells.

*RV, Rotavirus; FM-CBAL74, fermented milk Lactobacillus paracasei CBA L74; NFM, not fermented milk.*
